# Explaining the neural activity distribution associated with discrete movement sequences: Evidence for parallel functional systems

**DOI:** 10.3758/s13415-018-00651-6

**Published:** 2018-11-07

**Authors:** Willem B. Verwey, Anne-Lise Jouen, Peter F. Dominey, Jocelyne Ventre-Dominey

**Affiliations:** 1 Department of Cognitive Psychology and Ergonomics, University of Twente, Twente, The Netherlands; 2 Human Performance Laboratories, Department of Health and Kinesiology, Texas A&M University, College Station, TX USA; 3 INSERM U846, Stem Cell and Brain Research Institute, Bron, France

**Keywords:** Discrete sequence production task, Sequence learning, Execution modes, fMRI

## Abstract

To explore the effects of practice we scanned participants with fMRI while they were performing four-key unfamiliar and familiar sequences, and compared the associated activities relative to simple control sequences. On the basis of a recent cognitive model of sequential motor behavior (C-SMB), we propose that the observed neural activity would be associated with three functional networks that can operate in parallel and that allow (a) responding to stimuli in a *reaction mode*, (b) sequence execution using spatial sequence representations in a *central-symbolic mode*, and (c) sequence execution using motor chunk representations in a *chunking mode*. On the basis of this model and findings in the literature, we predicted which neural areas would be active during execution of the unfamiliar and familiar keying sequences. The observed neural activities were largely in line with our predictions, and allowed functions to be attributed to the active brain areas that fit the three above functional systems. The results corroborate C-SMB’s assumption that at advanced skill levels the systems executing motor chunks and translating key-specific stimuli are racing to trigger individual responses. They further support recent behavioral indications that spatial sequence representations continue to be used.

## Introduction

An important current research issue concerns the way in which people control habitual movement sequences like writing one’s signature and typing one’s name. Over the years, this issue has been addressed with numerous behavioral and imaging studies (for recent reviews, see Abrahamse, Ruitenberg, De Kleine, & Verwey, 2013; Ashby, Turner, & Horvitz, 2010; Diedrichsen & Kornysheva, 2015; Hardwick, Rottschy, Miall, & Eickhoff, 2013; Keele, Ivry, Mayr, Hazeltine, & Heuer, 2003; Penhune, 2013; Penhune & Steele, 2012; Verwey, Shea, & Wright, 2015). Meta-analyses of imaging studies show that motor control and motor learning are generally associated with increased activity in the primary motor cortex (M1), the dorsal premotor cortex, the primary somatosensory cortex (S1), the superior parietal lobule, the supplementary motor areas (SMAproper and preSMA), the putamen, the thalamus, and multiple cerebellar nuclei (Hardwick et al., 2013; Laird et al., 2011; Toro, Fox, & Paus, 2008). Laird et al. (2011, also see Ray et al., 2013) distinguished three motor networks with different functions: (a) a network including M1, S1, and the cerebellum that is responsible for executing hand and finger movements like finger tapping, grasping, and pointing; (b) the medial superior parietal cortex that extends this M1-S1-cerebellar network and that supports the execution of more complicated motor skills like drawing and reaching; and (c) a network consisting of premotor and supplementary motor cortices that is involved in preparing and executing fixed movement sequences and their timing. These meta-analyses synthesize the commonalities across many tasks, but they do not give detailed information on the functional contribution of each of these brain structures to motor behavior in specific tasks, and exactly how practice affects the associated neural activity patterns. For that reason, there is a need for studies addressing more specifically the function of individual brain regions in motor tasks.

Motivated by recent developments, we believe that a detailed understanding of the neural system requires insights from cognitive task models (Berlot, Popp, & Diedrichsen, 2018; Cookson, Hazeltine, & Schumacher, 2016; Forstmann, Wagenmakers, Eichele, Brown, & Serences, 2011; Krakauer, Ghazanfar, Gomez-Marin, MacIver, & Poeppel, 2018; Love, 2016). The reason is that these cognitive models distinguish separable processes that most likely emerge from activity in different neural networks. We report here an imaging study in which participants performed a *Discrete Sequence Production* (DSP) task (Verwey, 1999). This task is interesting for imaging research because extensive behavioral study has produced detailed cognitive models (Abrahamse et al., 2013; Verwey, 2001; Verwey et al., 2015). Also, unlike many other motor tasks, the DSP task lends itself to scrutiny in MRI scanners because it involves movements that give little motion artefacts.

Participants in DSP experiments practice two short (“discrete”) key-pressing sequences separated by a clear break. While practicing in an initial phase in which participants react to two series of key-specific stimuli, the task turns into a two-choice reaction time task in which each response consists of a familiar keying sequence. From a behavioral perspective these discrete movement sequences are interesting because the resulting motor representations are believed to produce the building blocks of complex, hierarchically controlled motor skills (Balleine, Dezfouli, Ito, & Doya, 2015; Cisek & Kalaska, 2010; Park, Wilde, & Shea, 2004; Shea & Kovacs, 2013; for recent real world examples, see Arnold, Wing, & Rotshtein, 2017; Thompson, McColeman, Stepanova, & Blair, 2017; Yamaguchi, Crump, & Logan, 2012). Recent interest comes from robot designers who are inspired by the way evolution shaped human motor control when they develop algorithms for motor learning and control in robots (Kupferberg et al., 2011; J. Peters, Mülling, Kober, Nguyen-Tuong, & Krömer, 2011).

### C-SMB: A cognitive model for sequencing behavior

In the present study we assessed the neural activity associated with the learning and execution of keying sequences in the DSP task, and we interpreted that activity in terms of the execution modes proposed by the *Cognitive framework for Sequential Motor Behavior* (C-SMB, Verwey et al., 2015; an extension of the *Dual Processor Model*, Abrahamse et al., 2013; Verwey, 2001) .^1^ This is a useful undertaking because C-SMB provides indications as to why imaging of different motor sequencing experiments usually shows a broad activation pattern that also greatly differs across tasks. C-SMB suggests that in the case of short, discrete movement sequences this is caused by a functional *Central Processor* and a *Motor Processor* that together are responsible for three sequence execution modes: (a) a *reaction mode* in which the Central Processor translates each key-specific stimulus into the associated response, (b) a *central-symbolic mode* in which within tens of trials responses are derived by the Central Processor from spatial and verbal sequence representations (representing, e.g., one’s PIN), and (c) a *chunking mode* in which after hundreds of trials motor chunk representations are used by the Motor Processor to rapidly execute the responses that make up the motor sequence. The reduced involvement of the Central Processor in the chunking mode explains the automatic nature of motor sequence execution and the limited role of explicit sequence knowledge in sequential motor skills (Baars, 1988, 2002). Motor chunks include only four or five key sequence elements (Acuna et al., 2014; Verwey, 2003; Verwey, Abrahamse, & De Kleine, 2010; Wymbs, Bassett, Mucha, Porter, & Grafton, 2012), so that longer sequences involve spontaneously developing concatenation of motor chunks. An important insight from behavioral findings is that at skilled levels the Motor Processor executing motor chunks may be racing with the Central Processor translating key-specific stimuli. Interestingly, recent findings suggest that spatial representations continue to be used for determining responses as well (Barnhoorn, Döhring, Van Asseldonk, & Verwey, 2016; Verwey, Groen, & Wright, 2016).

### Neural activity in the present study

We recently reported data from an fMRI study showing that timing in new (i.e., *unfamiliar*) four-key DSP sequences relies on a cortico-cerebellar network (Jouen et al., 2013). The present article used previously undescribed data from the same study in order to uncover networks associated with C-SMB’s reaction, central-symbolic, and chunking modes. To that end, we analyzed the blood oxygen level-dependent (BOLD) response, relative to simple control sequences, when unfamiliar and highly practiced (*familiar*) four-key sequences are being executed. On the basis of C-SMB, we propose that (a) the neural activity observed when unfamiliar sequences are executed reveals the neural substrate of reacting to key-specific stimuli as well as using (spatial) central-symbolic representations, and that (b) the neural activity during execution of the familiar sequences shows the neural areas involved in executing motor chunk representations and, again, reacting to key-specific stimuli. In turn, these two assumptions imply that (c) the activity overlap between unfamiliar and familiar sequences reflects the areas involved in reacting to key-specific stimuli. We note that C-SMB’s distinction between neural systems for central symbolic and motor chunk representations is similar to Hikosaka et al.'s (1999) spatial and motor neural networks, but unlike Hikosaka et al. we make the explicit assumption that these sequencing systems, and the system responsible for triggering individual responses using the element-specific stimuli, may be simultaneously active.

On the basis of imaging studies with choice reaction time tasks in which individual responses are given to stimuli, we expected that reacting to key-specific stimuli in the unfamiliar and familiar sequences would activate occipital and temporal areas for stimulus processing, right dorsal prefrontal and superior parietal areas for stimulus localization, and the left dorsal premotor cortex for selecting movements on the basis of stimulus location (Adam et al., 2003; Grafton, Fagg, & Arbib, 1998a, b; Picton et al., 2006; Rushworth, Johansen-Berg, Göbel, & Devlin, 2003; Schumacher, Elston, & D'esposito, 2003; Schwarb & Schumacher, 2009). Earlier work suggests that parietal activities may reflect spatial, but also non-spatial, response selection mappings (like category membership, Seger, 2008). Furthermore, the preSMA would be involved in storing and retrieving visuomotor associations during the selection of responses (Nakamura, Sakai, & Hikosaka, 1998; Picard & Strick, 2001; Sakai et al., 1999).

Executing unfamiliar sequences is typically associated with activity across a broad neural network. According to C-SMB, this network is involved in the spatial and/or verbal central-symbolic and the reaction modes, but does not involve activities associated with executing motor chunks. For these unfamiliar sequences, we expected activity in the well-known cortico-striatal-palidal-thalamo-cortical (in short: cortico-subcortical) executive loop that is engaged in working memory (Alexander, DeLong, & Strick, 1986; Lawrence, Sahakian, & Robbins, 1998; Seger, 2009). This loop comprises DLPFC (in the present task possibly responsible for explicit spatial element order; Bo, Peltier, Noll, & Seidler, 2011), bilateral posterior parietal cortex (coding spatial aspects of the responses and the sequence; Bo et al., 2011; Grafton, Hazeltine, & Ivry, 1998), the anterior basal ganglia (especially the associative striatum that would control element order; Bo et al., 2011; Doyon et al., 2009; Haber, 2003; Jueptner et al., 1997; Seger & Spiering, 2011), and several nuclei in the cerebellum (Bo et al., 2011; Floyer-Lea & Matthews, 2005; Hikosaka et al., 1999; Penhune & Doyon, 2005; Steele & Penhune, 2010; Wymbs & Grafton, 2014). SMA activity in sequencing tasks has further been associated with representing motor sequences in working memory (Cona & Semenza, 2017; Doyon et al., 2002; Halsband & Passingham, 1982; Jenkins, Brooks, Nixon, Frackowiak, & Passingham, 1994; Rosenberg Katz et al., 2012). PreSMA activity would be involved especially in early practice (Hill & Schneider, 2006), and would indicate temporal aspects of motor sequences (Cona & Semenza, 2017). Dorsal and ventral premotor cortex, along with cerebellum (especially lobule VI), would support initial sequence learning (Bo et al., 2011; Wymbs & Grafton, 2014). The activity usually observed in the left dorsal premotor cortex has been associated with retrieving sequence-specific, effector-unspecific knowledge (Bo et al., 2011; Gabitov, Manor, & Karni, 2016; Ohbayashi, Picard, & Strick, 2016), possibly in a spatial code (Nakamura et al., 1998). Instead, the activity often observed in the ventral premotor areas would represent processes critical for visually guided movements (Bisschoff-Grethe, Goedert, Willingham, & Grafton, 2004; Hoshi & Tanji, 2007; Mushiake, Inase, & Tanji, 1991; Werner, Dannenberg, & Hoffmann, 1997).

C-SMB further postulates that after substantial practice short motor sequences are primarily controlled using motor chunks. Based on imaging studies with extensively practiced sequences we hypothesized that the chunking mode would activate the cortico-subcortical sensorimotor loop that includes SMA and the posterior striatum (Hikosaka et al., 1999; Seger, 2009). At this practice level, SMAproper has been argued to develop and implement motor chunks (Cona & Semenza, 2017; Verwey, Lammens, & van Honk, 2002), and preSMA would initiate individual motor chunks (Kennerley, Sakai, & Rushworth, 2004; Ruitenberg, Verwey, Schutter, & Abrahamse, 2014; Shima & Tanji, 1998). Should spatial representations indeed be used at advanced skill levels too (Barnhoorn et al., 2016; De Kleine & Verwey, 2009; Verwey et al., 2016), then activity can be expected in the left dorsal premotor and parietal cortices too (Nakamura et al., 1998; Ohbayashi et al., 2016).

In summary, we hypothesized that: (1) Executing relatively unfamiliar keying sequences activates a broad network underlying both the reaction and central-symbolic modes. Sequence execution on the basis of spatial central-symbolic sequence representations was expected to induce frontal activity (especially in DLPFC, dorsal and ventral premotor cortex, and SMA), bilateral posterior parietal activity, and activity in the anterior basal ganglia and cerebellar nuclei. (2) Executing familiar sequences was expected to activate a network involved in especially the chunking mode, which would include the SMA and posterior striatum, possibly extended by activity of the left dorsal premotor and some parts of the parietal cortex to support the reaction mode. Finally, (3) given the assumption that stimuli may still trigger individual responses in both unfamiliar and familiar sequences, we expected activity across the unfamiliar and familiar sequences in frontal areas (the right dorsal prefrontal and left dorsal premotor cortex, and preSMA), superior parietal areas, and temporal/occipital areas (see Table 3 for an overview of predictions and observed activities).

## Method

### Participants

Eighteen right-handed healthy volunteers participated in this study (mean age 22.5 years, SD=1.8; eight males). The participants were all students from Lyon University. Prior to the scanning session, they underwent an examination to validate their medical state and MRI compatibility. No participant had a history of neurological no psychiatric disorders. They all completed the entire fMRI test. Two of them were removed from the analysis because of the high number of motion-related artefacts in the cerebral images. The protocol was approved by the Lyon Ethics Committee (Centre Léon Berard CPP number: 06/013) and the participants gave their informed consent before the scanning session.

### Apparatus

The experimental protocol was implemented in Presentation (Neurobehavioral Systems, Albany, NY, USA) on a Windows XP-based PC that also measured the four response times (RTs) in each sequence (i.e., T_1–_T_4_) in both sessions. A Lumina key pad (Cedrus, San Pedro, CA, USA) was used for registering key presses that are suited for use in MRI scanners. The RTs and errors were analyzed using Statistica (Statsoft Inc., Tulsa, OK, USA). fMRI was assessed with a 1.5 T system (Siemens CTI, Ann Arbor, MI, USA) at the Imaging Center of Lyon (CERMEP “Imagerie du vivant”).

### Task and stimuli

While performing the DSP task, participants rested four fingers of the right hand on four keys of the key pad. Visual stimuli displayed on the screen involved filling one of four permanently displayed squares. The participants responded to these stimuli by pressing the spatially corresponding key. As soon as the correct key had been pressed, the square was filled again with the background color and immediately another square was filled until four keys had been pressed. The participants were instructed to press the associated key as fast as possible while keeping errors to a minimum. Faulty key presses were immediately followed by an error message (by changing the visual stimulus from white to a color). This DSP task has previously been described more eloborately (Verwey, 1999; for a review of method and results, see Abrahamse et al., 2013).

We used four experimental sequences: IRML, MLIR, RIML, and LMRI (Index, Middle, Ring, Little finger), and two simple control sequences: IMRL, LRMI. The experimental sequences never involved key presses by adjacent fingers while the control sequences involved an order that was easy to learn in that it included a left-to-right or a right-to-left succession of the four key presses. These six sequences were divided in participant-specific ways into a familiar and an unfamiliar set, each one consisting of one control sequence and two experimental sequences. One of the two experimental sequences in both the familiar and unfamiliar sets was structured by including an 800-ms pause between the second response and the third stimulus, while all other RSIs were 0. In the structured sequence of the familiar set this pause occurred during practice but not during scanning, while in the unfamiliar-structured sequence it occurred during scanning. Because in the present study we focused on the unstructured sequences, any mention of unfamiliar and familiar sequences in his article refers to the unfamiliar-unstructured and familiar-unstructured sequences. The experimental sequences were balanced so that, across all participants, each of the four experimental sequences occurred as frequently in each of the four experimental conditions (familiar and unfamiliar sequences, without and with a pause). Likewise, the two control sequences were evenly distributed across the familiar and unfamiliar sequence sets.

The experiment included a 14-block practice session that was followed after a 30-min break by a fMRI scanning session. During practice, participants sat in front of a computer display on a table with the fingers of their right hand on a key pad. Each practice block included the three sequences of the familiar set (structured, unstructured, and control) in a random order. These sequences were practiced for a total of 1,500 trials, approximately 500 trials for each sequence. A 10-min pause was inserted halfway through the practice session. The entire practice session lasted approximately 2.5 h.

### fMRI scanning

#### Setup

At the start of the scanning session, participants were comfortably installed in the MRI scanner. Head movements were prevented using a foam cushion and a frontal band, which were attached to the scanner bed. The visual stimuli were displayed by video-projector on a translucent screen located behind the scanning bay. The participant looked at the screen via a mirror fixed inside the scanner at 20 cm over the participant’s head. The key pad was located comfortably on the participant’s lap.

### Scanning procedure

Brain scanning involved assessment of the BOLD fMRI signal. For each run, whole brain coverage was obtained with EPI (echo planar imaging) images (repetition time TR=2,500 ms, echo time TE=60 ms, and flip angle 90°). Twenty-six brain sections were acquired in an interlaced mode parallel to the AC-PC plane. Slices had a thickness of 4.4 mm [matrix 64 × 64; and field of view (FOV) = 230 mm]. Following functional image acquisition, a high-resolution T1-weighted anatomic image was acquired (TR=1,880 ms; TE 3.93; flip angle 15°; matrix 256 × 256; and slice thickness 1 mm).

The scanning session involved four runs. Two runs included three unfamiliar sequences: one experimental sequence that included an 800-ms pause between the second response and the ensuing stimulus during scanning, one experimental sequence without pause, and one control sequence. The other two runs included three familiar sequences: two experimental sequences without a pause and one control sequence. These three sequences had been practiced prior to scanning, but the pause between the second response and the third stimulus in one of them was removed during scanning. Half the participants started the scanning session with a familiar sequences run, the other half with an unfamiliar sequences run. Then familiar and unfamiliar runs alternated. Each run lasted approximately 10 min and successive runs were separated by 2-min breaks. Each run included 150 trials, 50 trials with each of the three sequences. These three sequences were executed in random order. The interval between the first stimulus of two successive sequences was 4 s in duration, jittered by trials of 8-s ISI.

### fMRI data analysis

Processing of the fMRI data involved Statistical Parametric Mapping software (SPM 5, Welcome Department of Imaging Neuroscience, London UK; http://www.fil.ion.ucl.ac.uk/spm) running under Matlab (The Mathworks, Inc., Natick, MA, USA). The first five scans of each run (i.e., the first 12.5 s and hence the first two or three sequences) were discarded to eliminate non-equilibrium effects of magnetization and warming up effects in the participants. For preprocessing, the functional images were realigned with respect to the first functional image for motion correction and were corrected for slice acquisition timing in reference to the middle slice in each scan. The resulting volumes were spatially normalized to fit to an EPI template in MNI (Montreal Neurological Institute) space. The normalized images were then spatially smoothed using an isotropic Gaussian filter kernel at an 8-Hz bandwidth. For each participant the BOLD impulse responses to different event types were modeled in the context of a general linear model (GLM) by using the hemodynamic response function (HRF) convolved with a delta (event-related) function.

On the basis of the GLM model (Friston et al., 1994), the task-related BOLD changes were estimated as linear combinations of the individual regressors and stored as participant-specific contrast images. Contrasts were realized using several regressors in order to study the differences between the experimental and control sequences (i.e., unfamiliar-experimental, unfamiliar-control, familiar-experimental, familiar-control). This involved unfamiliar-experimental > unfamiliar-control and familiar-experimental > familiar-control. The overall contrasts included the following four contrasts: unfamiliar-unstructured > unfamiliar-control, familiar-unstructured > familiar-control. These contrasts were selected to extract the activated neural structures that were differently activated when executing the familiar and unfamiliar unstructured experimental sequences relative to the control sequences.

For the statistical group analysis, the individual contrast images were then processed in a second-level random effects model by using a full factorial design to extract significant neural activations for each type of sequence. The main effects for learning unstructured sequences were processed and specific activations related to familiar and unfamiliar sequences were extracted by inclusive masking at p<.05 with the respective t-contrasts, i.e., familiar-unstructured > familiar-control and unfamiliar-unstructured > unfamiliar-control. Significance level of activation was established with a false discovery rate (FDR) correction at the voxel level (threshold of p<.005) for whole-brain voxels with minimal spatial extent of 10 contiguous voxels per cluster. In order to determine the neural structures activated in common during the familiar and unfamiliar unstructured sequences, we performed a conjunction analysis between the corresponding contrasts at p_uncorrected_<.001, based on Nichols’ procedure (Nichols, Brett, Andersson, Wager, & Poline, 2004). All MNI coordinates of the cerebral activation foci were transformed into Talairach coordinates using the formula developed by Matthew Brett (http://imaging.mrc-cbu.cam.ac.uk/imaging/MniTalairach). Brodmann areas were determined using the stereotaxic atlas (Talairach & Tournoux, 1988).

## Results

### Behavioral results

#### Response times

**Fig. 1:**
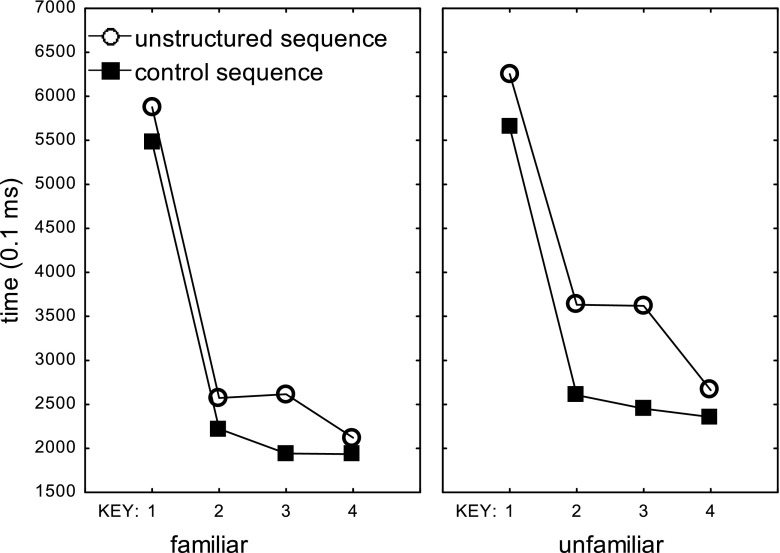
Response times in the familiar and unfamiliar and control sequences across sub-runs 1–4 in the test phase carried out in the scanner

We analyzed RTs in the scanning session with a 2 (Familiarity) × 2 (Type: unstructured vs. control) × 4 (Sub-run: Trials 1–25 vs. 26–50 of Runs 1 and 2) × 4 (Sequence Position T_1_–T_4_) repeated measures ANOVA. This analysis showed main effects of Familiarity, F(1,15)=41.1, p<.001, *η*_*p*_^*2*^=.73, Type, F(1,15)=89.8, p<.001, *η*_*p*_^*2*^=.86, Sub-run, F(3,45)=16.8, p<.001, *η*_*p*_^*2*^=.53, and Key, F(3,45)=175.9, p<.001, *η*_*p*_^*2*^=.92. As expected, the difference between the unstructured and control sequences was larger in the unfamiliar than in the familiar condition, F(1,15)=4.9, p=.04, *η*_*p*_^*2*^=.25. Practice in the four sub-runs reduced T_2_-T_4_ more than T_1_, F(9,135)=16.7, p<.001, *η*_*p*_^*2*^=.53 (see Figure 1).

#### Errors

A 2 (Familiarity) × 2 (Type: unstructured, control) × 2 (Run 1 vs. 2) × 4 (Sequence Position) repeated measures ANOVA was carried out on arcsine transformed error proportions. The Key main effect showed that error rate increased from 1.0% at Response 1, to 1.8% at Response 3, and reduced to 0.4% at Response 4, F(3,45)=9.4, p<.001, *η*_*p*_^*2*^=.39. This relatively high error rate at Response 3 was more pronounced for the familiar than for the unfamiliar sequences, 2.3% versus 1.3%, F(3,45)=4.5, p<.001, *η*_*p*_^*2*^=.23, and higher for the control than for the unstructured sequences, 2.5% versus 1.0%, F(3,45)=4.2, p=.01, *η*_*p*_^*2*^=.22.

### Functional MRI results

In the analyses below, we removed movement execution-related activities by using contrasts relative to the left-to-right and right-to-left control sequences, and focused on sequence learning by determining activations of the familiar and unfamiliar sequences.

#### Unfamiliar sequences

**Fig. 2:**
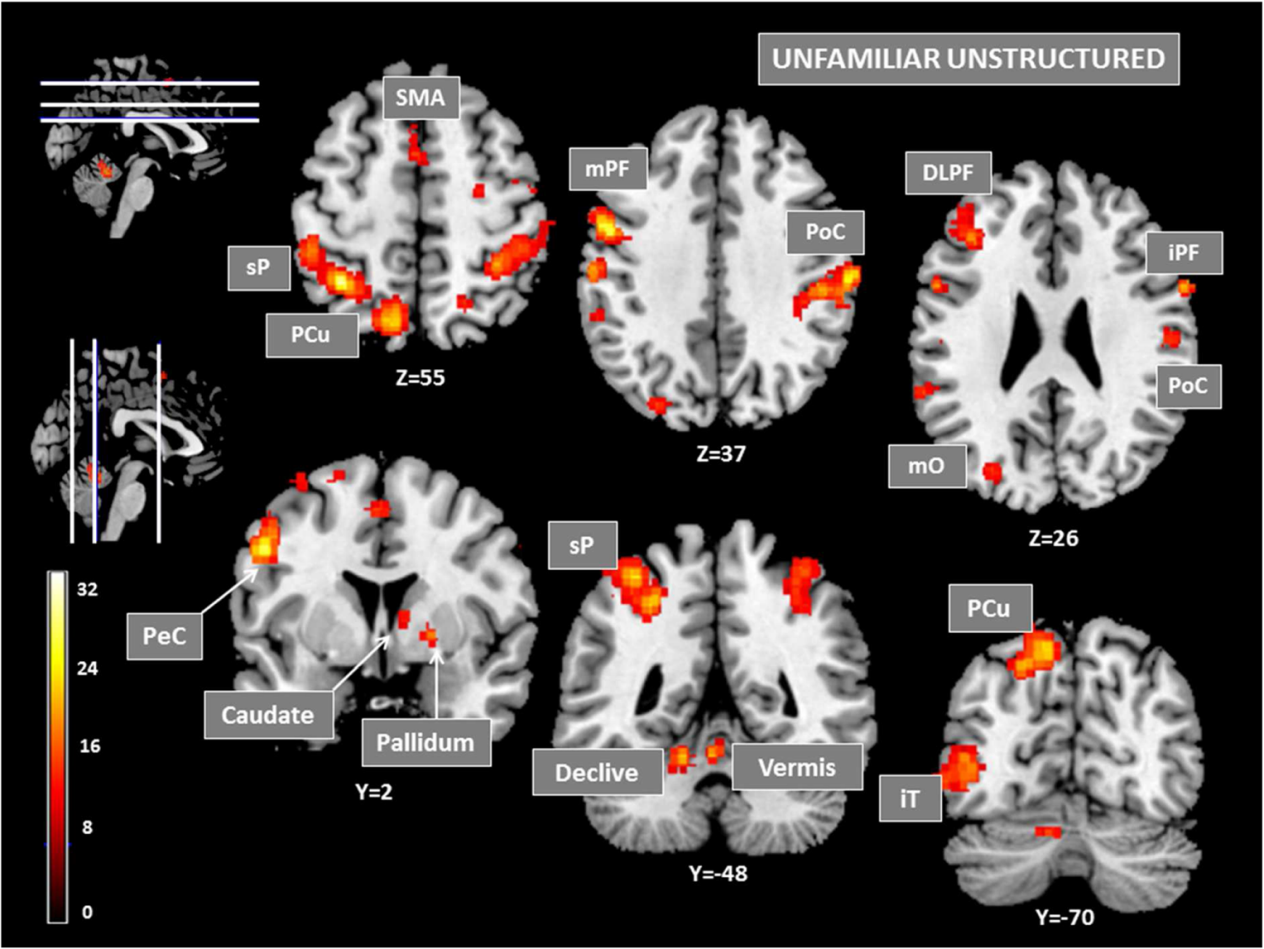
Clusters of activation for the unfamiliar sequences (unfamiliar-unstructured>control sequences) at FDR p_corr_< .05. Activations are displayed on serial transverse (upper part) and coronal (lower part) sections in the stereotaxic space of Talairach and Tournoux (1988) with slice locations indicated below each image. On the left: level of the sections on lateral views of the brain and scale of the t values. *SMA* supplementary motor area, *sP* superior parietal cortex, *PCu* precuneus, *PoC* postcentral cortex, *PeC* precentral cortex, *mPF* middle prefrontal cortex, *iPF* inferior prefrontal cortex, *DLPF* dorsoLateral prefrontal cortex, *mO* middle occipital cortex, *iT* inferior temporal cortex

Activity associated with unfamiliar sequences (revealed by the inclusive masking of unfamiliar sequences on the main effect of learning) appeared widespread across the brain (Table 1, Fig. 2). Anteriorly, significant BOLD changes were found bilaterally in the supplementary motor area (SMA) forming a spreading cluster peaking on the left side (Ke=91). The frontal lobe was strongly activated predominantly in the left hemisphere forming large clusters in (1) the superior frontal and precentral gyrus (BA6) symmetrically activated in both hemispheres, (2) the left middle prefrontal gyrus (BA6) extending to the precentral cortex posteriorly and including anteriorly the dorsolateral prefrontal cortex (DLPFC: BA9, 46) with a maximal activation at TAL -38 30 21 (Ke=261), and (3) the inferior prefrontal gyrus as a large left cluster spreading from the middle to the inferior prefrontal cortex (BA9, 6, 44) and as a right cluster peaking at TAL 61 9 27. Activity was further found bilaterally in the post-central sensorimotor cortex partially including the motor cortex (BA3, 4).

**Table 1.** Anatomical and functional regions of activation for unfamiliar and familiar sequences (both relative to the control sequences)

**Anatomic area** **(functional area** ^**1**^ **)**	**BA**	**Unfamiliar sequences**			**Familiar sequences**		
		**Tal**	**Z**	**Ke**	**Tal**	**Z**	**Ke**
B (pre)SMA	6	-2 6 49	3.77	91	-2 5 50	3.67	25
L Middle frontal gyrus L Precentral gyrus (L PMd)	6	-24 -7 46	3.78	29			
R Superior frontal gyrus R Middle frontal gyrus R Precentral gyrus (L PMd)	6				38 -4 65	3.87	72
					38 -3 46	3.30	
					42 -5 56	3.28	
		26 -9 50	3.45	29	24 -5 61	3.09	18
		24 -5 61	3.09				
		28 -4 70	3.08	18	28 -4 70	3.08	
L Superior frontal gyrus (L PMd)	6	-32 -1 59	3.44	98	-32 -1 59	3.44	30
		-20 -8 66	3.40				
L Middle frontal gyrus L Precentral gyrus (L PMv/PMd)	6,9, 44	-50 4 35	4.91	428			
		-53 9 23	4.09				
L Inferior frontal gyrus (L PMv)		-47 2 42	4.08				
R Inferior frontal gyrus R Precentral gyrus (R PMv)	6, 9	61 9 27	4.42	142			
		63 0 37	3.50				
L Middle frontal gyrus (L DLPFC)	9,45,46	-38 30 21	4.32	261			
		-40 38 20	3.53				
		-34 29 30	3.26				
R Middle frontal gyrus	46	44 47 16	3.56	24			
L Postcentral gyrus L Precentral gyrus (L S1/M1)	4,3	-57 -16 32	4.21	108			
R Postcentral gyrus (R S1) R Inferior parietal gyrus	4,2,3,5, 40	53 -23 40	5.26	1851	40 -31 38	4.14	543
		63 -18 34	4.67		34 -40 55	4.05	
		34 -40 52	4.41		42 -30 49	3.82	
R Superior parietal gyrus R Precuneus	7	18 -55 52	3.73	55	20 -57 52	3.47	36
		26 -55 60	3.08		26 -55 60	3.08	
		20 -49 65	3.40	63	20 -49 65	3.40	63
L Superior parietal gyrus L Inferior parietal gyrus L Precuneus	40, 7	-28 -39 42	5.20	2117	-34 -46 56	5.16	894
		-34 -46 56	5.16		-46 -36 48	4.45	
		-20 -64 47	4.71		-12 -65 55	4.42	
L Middle occipital gyrus L Inferior temporal gyrus	37, 39	-42 -66 7	4.35	384			
		-53 -68 1	3.76				
L Middle occipital gyrus L Fusiform gyrus	19,37	-44 -61 -14	3.17	15			
		-46 -62 -6	3.07				
R Superior temporal gyrus	38				40 18 -29	3.89	40
R Pallidum-putamen		22 2 0	3.97	29			
R Caudate nucleus (anterior)		12 2 7	3.46	48			
R Striatum (posterior)		8 8 -2	3.43		24 -34 13	3.63	21
		6 -3 11	3.00				
L Cerebellum – declive, culmen		-14 -47 -13	4.37	327			
		-2 -47 -13	4.33				
		-24 -40 -23	4.19				
L Cerebellum – declive		-8 -67 -19	3.82	40			

*Notes.* FDR correction at p < .05

*B* bilateral, *R* right, *L* left, *Tal* Talairach coordinates, *BA* corresponding Brodmann’s area

Functional area designations (in parenthesis) are based in part on Mayka, Corcos, Leurgans, and Vaillancourt (2006) and the Talairach client (Lancaster et al., 1997; Lancaster et al., 2000). We focused here on the unstructured sequences

Posteriorly, extrastriate cortical areas were also activated during execution of the unfamiliar sequence mainly in the left hemisphere, with important BOLD changes (Ke=2117) peaking at TAL -28 -39 42 in the inferior parietal cortex (BA40) extending medially to the precuneus (BA7). As illustrated in Fig. 2, a substantial cluster was identified in the left temporo-occipital region including associative visual areas (BA19, 37, 39). In the subcortical structures, executing the unfamiliar sequences significantly activated the basal ganglia at two small right patches in the postero-ventral region of the putamen and caudate nucleus, and a substantial left activation in the cerebellum that formed a large cluster (Ke 327) peaking in the declive at Tal -14 -47 -13 and extending medially over 20 mm to the vermis.

#### Familiar sequences

**Fig. 3:**
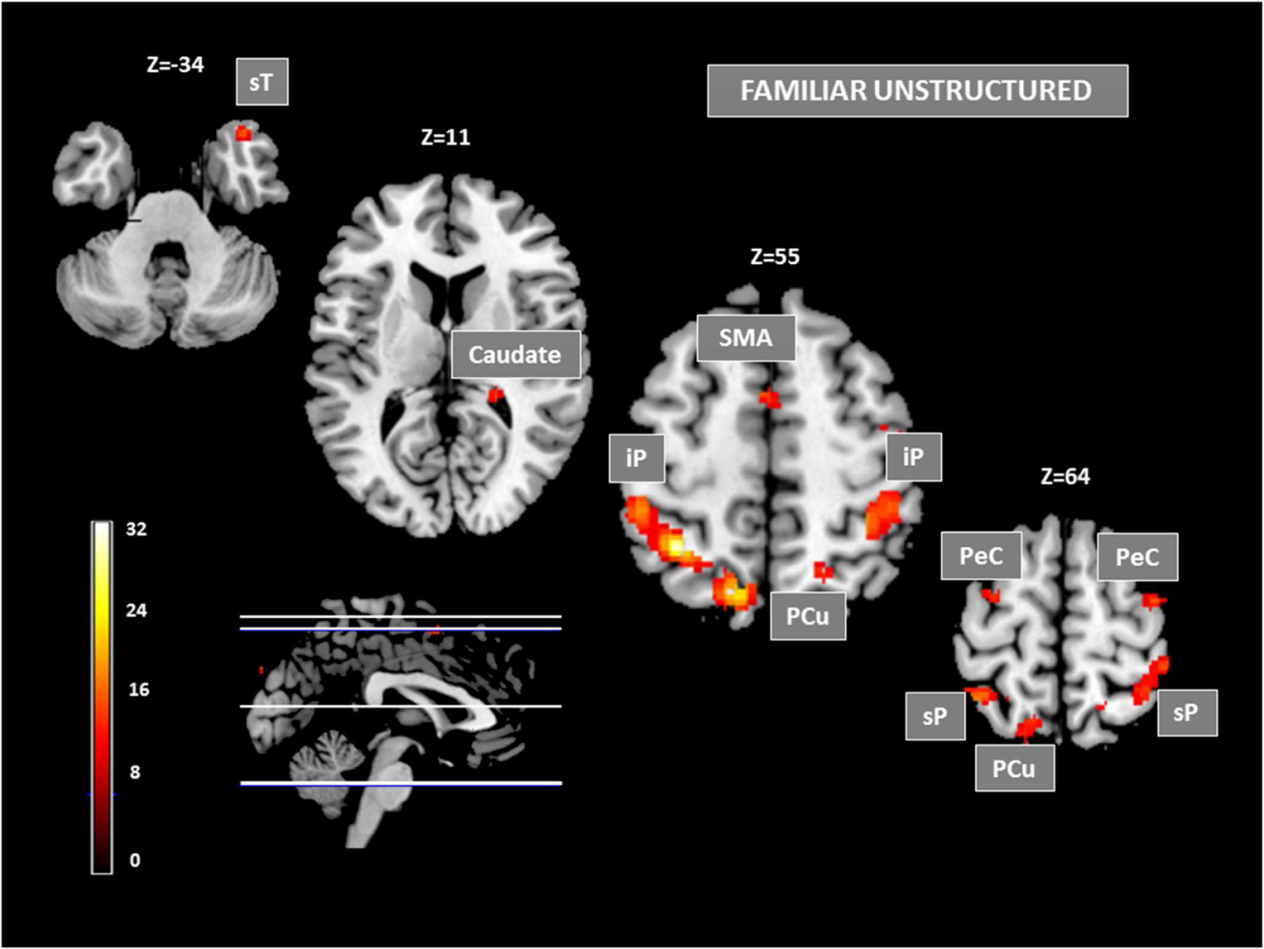
Clusters of activation for the familiar sequences (familiar-unstructured>control sequences) at FDR p_corr_< .05. Activations are displayed on serial transverse sections in the stereotaxic space of Talairach and Tournoux (1988) with slice locations indicated over each image. At the left: level of the sections on a lateral view of the brain and scale of the t values. *sT* superior temporal cortex, *SMA* supplementary motor area, *iP* inferior parietal cortex, *PCu* precuneus, *PeC* precentral cortex, *sP* superior parietal cortex

Cerebral activation associated with familiar sequences (revealed by inclusive masking of familiar sequences on the main effect of learning) was globally reduced relative to unfamiliar sequences, particularly in the prefrontal cortex, as shown in Fig. 3 and Table 1. Importantly, while the inferior and middle prefrontal cortices including DLPFC were no longer recruited, the SMA and the superior and middle prefrontal cortex (BA6) remained activated, forming several bilateral small clusters (Table 1). In the posterior cortex, substantial BOLD responses were found bilaterally in the superior and inferior parietal cortex (BA7, 40) forming large clusters extending medially and caudally over 10 mm into the precuneus on the left and the supramarginal region on the right. While the middle occipital regions were not recruited with familiar sequences, the right superior temporal cortex was found activated in the temporal pole (BA 38) as a limited focus peaking at TAL 40 18–29. In the subcortical region, a significant BOLD response was observed in basal ganglia only in the right caudate nucleus.

#### Across unfamiliar and familiar sequences

**Fig. 4:**
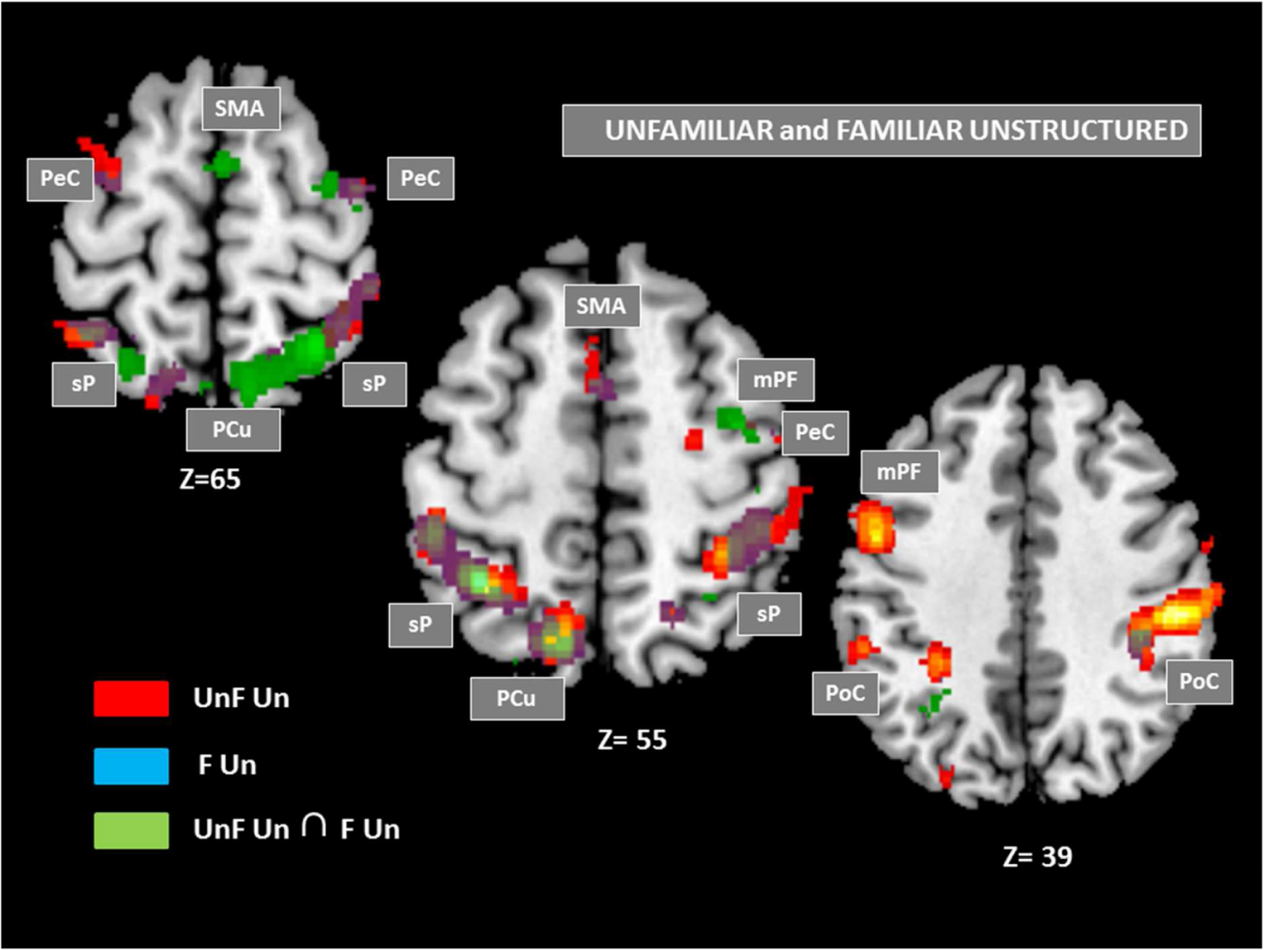
**C**lusters of activation for the (unstructured) sequences as red clusters for unfamiliar-unstructured>control sequences (UnF Un), blue clusters for familiar-unstructured>control sequences (F Un) at FDR p_corr_< .05 and green clusters for the significant common activation issued from the conjunction statistical analysis (UnF Un ∩ F Un) at p_unc_ < .001. **P**urple represents areas where there was significant activation in Unf Un (red) and significant activation of F Un (blue), but where the statistical measure of conjunction was not significant at p(unc) < .001. Activations are displayed on serial transverse sections in the stereotaxic space of Talairach and Tournoux (1988) with slice locations indicated below each image. *SMA***s**upplementary **m**otor area**,***sP***s**uperior **p**arietal cortex**,***PCu***p**recuneus**,***PoC***p**ostcentral cortex**,***PeC***p**recentral cortex**,***mPF***m**iddle **p**refrontal cortex

By using a conjunction analysis that provides the common activated network for familiar and unfamiliar sequences, we found that executing both these sequences activated a substantial network of areas, including the SMA (with a peak in preSMA), two right dorsal premotor areas (BA6), a large cluster extending bilaterally in the superior parietal lobule (BA7) and precuneus up to the inferior parietal cortex on the left (BA40). The statistical data related to these activations are shown in Table 2. Fig. 4 illustrates the activation in common for the unfamiliar and familiar sequences along with the specific activation for each of them, unfamiliar and familiar sequences.

**Table 2.** Anatomic and functional regions of significant activation, relative to the control sequences, in common for familiar and unfamiliar sequences (i.e., the conjunction analysis)

**Anatomic area** **(Functional area** ^**1**^ **)**	**BA**	**Unfamiliar and familiar sequences**		
		**Tal**	**Z**	**Ke**
B (pre)SMA	6	4 3 57	3.80	53
R Superior-middle frontal gyrus R Precentral gyrus (R PMd)	6	40 -7 56	4.22	121
		28 -10 70	3.67	54
R Superior parietal gyrus R Precuneus	7	28 -51 65	5.23	577
		14 -57 67	4.01	
		10 -47 69	3.83	
L Superior parietal gyrus L Inferior parietal gyrus L Precuneus	7, 40	-34 56 45	3.65	69
		-28 -50 39	3.37	
		-26 -55 60	3.71	52
		-28 -67 47	3.54	20

*Tal* Talairach coordinates, *BA* corresponding Brodmann’s area

Functional area designations (in parenthesis) are based in part on Mayka et al. (2006) and the Talairach client (Lancaster et al., 1997; Lancaster et al., 2000)

## Discussion

The cognitive C-SMB model hypothesizes that separable functional networks are responsible for learning discrete keying sequences in terms of spatial and motor coordinates, while in addition key-specific stimuli continue to be translated into individual responses. As systematically shown in Table 3, the regional activities that we derived from the literature using this hypothesis appeared in good alignment with the observed activities relative to the control sequences, and they also provided support for recent behavioral indications that spatial sequence knowledge continues to be used as skill develops.

**Table 3.** Overview of the predicted and observed relative neural activities with the presumed cognitive functions

**Speculative function**	**Unfamiliar**		**Unfamiliar and familiar** ^**1**^		**Familiar**	
	**predicted**	**observed**	**predicted**	**observed**	**predicted**	**observed**
Visuomotor association			preSMA	preSMA		
Loops, motor sequencing	preSMA; SMAproper	preSMA			preSMA; SMAproper	preSMA
R selection – stimulus guided	PMv	B PMv				
R selection –stimulus and representation guided	PMd	B PMd	L PMd	R PMd	L PMd	B PMd
Executive control, sequence detection	DLPFC	DLPFC	R DLPFC			
Movement execution		B S1; L M1				R S1
Stimulus location; spatial representation	B posterior parietal^2^	B posterior parietal; B precuneus	superior parietal	B superior parietal; B precuneus; L inferior parietal	B posterior parietal	B posterior parietal; B precuneus
Stimulus identification; visual seq. representation		L occipital; L inferior temporal; L fusiform	occipital, temporal			R superior temporal
Executive loop (control, WM)	anterior basal ganglia	R mid-ventral putamen R anterior caudate				
Sensorimotor loop (motor sequences)					posterior striatum	R posterior striatum
Sequence timing, feedback model	cerebellum-lobules V, VI, VIIB, VIII	L cerebellum-lobules V, VI				

^1^Overlapping activities in familiar and unfamiliar sequences include the conjunction analysis results in Table 2 as well as activities identical for unfamiliar and familiar sequences in Table 1

^2^Posterior parietal includes superior and inferior parietal areas

### Unfamiliar sequences

The RT results of the unfamiliar sequences show the typical slow first response followed by much faster execution of the ensuing responses. This RT pattern is not observed with sequences consisting of random stimulus orders (Barnhoorn, Panzer, Godde, & Verwey, 2018; Verwey & Wright, 2014). Performance therefore demonstrates that participants had started learning the unfamiliar sequences during the scanning session.

The neural activity pattern demonstrates that executing unfamiliar sequences activated bilateral DLPFC and anterior parts of the striatum. These two areas are parts of the executive cortico-subcortical loop that is involved in working memory (Haber, 2003; Lawrence et al., 1998; Seger & Spiering, 2011). Working memory is used when the links between sensory inputs, thoughts and actions are weak or rapidly changing (Miller & Cohen, 2001; Postle, 2006). In line with this idea, the DLPFC previously showed activation early in the course of sequence practice, and also when participants were instructed to again pay attention to the execution of an already familiar motor sequence (Jueptner et al., 1997), because DLPFC is associated with the spatial aspects of reacting in keying sequences (Robertson, Tormos, Maeda, & Pascual-Leone, 2001). We further observed activity in the right inferior frontal gyrus. This area would underlie the executive functions of total response inhibition, response-specific inhibition, and response delay (Aron, Robbins, & Poldrack, 2014). DLPFC most likely does this by modulating connectivity between other brain regions (Kübler, Dixon, & Garavan, 2006; Passingham, Rowe, & Sakai, 2013; Rae, Hughes, Anderson, & Rowe, 2015). Here, the right inferior frontal gyrus may have reduced the contribution of the reaction mode network as the central-symbolic execution mode developed.

The observed activities in the left occipital and left inferior temporal areas are typically associated with perceiving and identifying visual objects, and retaining these in working memory (Ishai, Ungerleider, Martin, Schouten, & Haxby, 1999; Kolb, Whishaw, & Teskey, 2014; Nakamura et al., 2000). The concurrent activity in the posterior parietal cortex and precuneus can be attributed to the orienting of overt and covert spatial attention (Giesbrecht, Woldorff, Song, & Mangun, 2003; Just & Varma, 2007). The repeated execution of fixed spatial attention patterns during sequence execution may have been responsible for the development of spatial central-symbolic sequence representations in the posterior parietal cortex and precuneus (Abrahamse et al., 2010; Zhang & Ekstrom, 2013).

The activity of the ventral part of the premotor cortex was specific for unfamiliar sequences. The ventral premotor cortices have been argued to translate allocentric perceptual coordinates into egocentric motor coordinates (Bisschoff-Grethe et al., 2004; Hoshi & Tanji, 2007; Mushiake et al., 1991; Werner et al., 1997), and they did indeed appear active especially early in practice (Toni, Rushworth, & Passingham, 2001). In the present unfamiliar sequences, the ventral premotor cortices may have selected responses on the basis of the stimulus locations they received from parietal areas that were developing a spatial central-symbolic sequence representation.

Executing unfamiliar sequences was further associated with activity in the left inferior frontal gyrus and the preSMA, areas known to play a domain-independent role in sequence control. The left inferior frontal gyrus overlaps in part with Broca’s area, and has been argued to not only be involved in verbal but also in spatial sequence representations (Binkofski & Buccino, 2006; Friederici, Rueschemeyer, Hahne, & Fiebach, 2003; Koechlin & Jubault, 2006). The preSMA, together with the inferior frontal left cerebellar and premotor cortex, is perhaps involved in the timing of motor chunk development (Cona & Semenza, 2017).^2^ Such a timing function would fit the left cerebellar activity observed by us and in other studies with unfamiliar sequences (Doyon et al., 1997; Doyon, Penhune, & Ungerleider, 2003; Jouen et al., 2013; Jueptner et al., 1997; Steele & Penhune, 2010).

In summary, the pattern of activity associated specifically with the unfamiliar sequences can be interpreted in terms of: (1) a prefrontal-striatal network that is at the basis of executive control of various functional processes including working memory, (2) a left occipital-left temporal-bilateral posterior parietal network for developing and applying central-symbolic (most likely spatial) sequence representation, (3) ventral premotor activity for selecting individual responses from the parietal sequence knowledge, and (4) a left inferior frontal-preSMA-cerebellar network responsible for timing of sequence execution.

### Familiar sequences

The notion that motor chunk development relies primarily on the cortico-subcortical sensorimotor loop (Cona & Semenza, 2017; Kennerley et al., 2004; Ruitenberg et al., 2014; Shima & Tanji, 1998; Verwey et al., 2002) is corroborated by the observed activity in the preSMA and the activity shift with practice in the posterior direction in the basal ganglia. This increasing role of motor chunks further fits the often encountered brain-wide activity reduction with practice, which can be seen in Table 1 as well (Chein & Schneider, 2005; Hill & Schneider, 2006; Picard, Matsuzaka, & Strick, 2013; Wymbs & Grafton, 2014).

Execution of familiar sequences was further associated with activity in the right superior temporal, bilateral posterior parietal, and dorsal premotor cortices (Table 1, cf. Bisschoff-Grethe et al., 2004; Wymbs & Grafton, 2013). The lasting activity in the dorsal premotor cortex, which coincided with the lasting activity in parietal areas, supports the idea that the dorsal premotor cortex receives spatial information from the parietal and the prefrontal cortices allowing it to select responses on the basis of parietal sequence representations (Chouinard & Paus, 2006; Rijntjes et al., 1999; Schwarb & Schumacher, 2009; Wiestler, Waters-Metenier, & Diedrichsen, 2014). These activities therefore support the suggestion in the *Introduction*, based on imaging studies (Nakamura et al., 1998; Ohbayashi et al., 2016) and behavioral DSP studies (Barnhoorn et al., 2016; De Kleine & Verwey, 2009; Verwey et al., 2016), that after extensive practice spatial sequence representations may still be involved in triggering the responses in the familiar sequences. This may especially occur when sequences are short, like the ones in the present experiment (Ellenbuerger, Boutin, Blandin, Shea, & Panzer, 2012; Panzer, Krueger, Muehlbauer, Kovacs, & Shea, 2009).

In short, execution of the familiar sequences in the chunking mode was associated with the sensorimotor loop that included the posterior striatum and SMA. The present results further indicated involvement of a network including areas in the temporal, posterior parietal, and dorsal premotor cortex that probably underlay continued involvement of the spatial central-symbolic execution mode.

### Reacting to stimuli

The areas showing activity in both unfamiliar and familiar sequences included preSMA, bilateral dorsal premotor areas, and bilateral posterior parietal and precuneus. As argued in the *Introduction*, we attribute these activities to responding to individual stimuli using spatial and perhaps also non-spatial associations (Seger, 2008). The preSMA activity common to unfamiliar and familiar sequences occurred about 10 mm from the activity associated exclusively with the unfamiliar and with the familiar sequences, suggesting the preSMA was involved in two different networks (Xiong et al., 2000). One network would be responsible for skilled motor sequence control (Cona & Semenza, 2017; Verwey et al., 2002), and the other for establishing and retrieving modality- and effector-independent visuomotor associations when responding to key-specific stimuli in the reaction mode (Nakamura et al., 1998; Picard & Strick, 2001; Sakai et al., 1999).

Reacting to stimuli seems to have also activated the dorsal premotor areas, the bilateral posterior parietal cortex, and the precuneus. In this network, the rostral part of the dorsal premotor cortex may well receive spatial stimulus information from prefrontal cortex and preSMA to select individual responses (Chouinard & Paus, 2006; Schwarb & Schumacher, 2009). The fact that we did not observe activity in the left occipital and inferior temporal areas across unfamiliar and familiar sequences is inconsistent with stimuli being reacted to at all practice levels. This may, however, been concealed because participants probably processed stimuli in our control sequences too (given that display of stimuli at different locations attracts visual attention; Proctor, Miles, & Baroni, 2011; Yantis & Jonides, 1984).

### Unanticipated activities

We did not anticipate the activity in S1 and left M1 when unfamiliar sequences were executed because all activities were relative to control sequences. This finding allows some speculation, however. Recent approaches assume that M1 codes movements in terms of highly familiar, meaningful behaviors like hand postures (Ejaz, Hamada, & Diedrichsen, 2015; Graziano, 2016; A. J. Peters, Lee, Hedrick, O'Neil, & Komiyama, 2017). The present M1-S1 activity may therefore reflect learning new hand postures, and/or new sequences of already familiar hand postures. Given the effector-specificity of M1 and S1, this early M1-S1 activity may eventually be responsible for the development of effector-specific sequence learning (Hikosaka et al., 1999; Verwey, Abrahamse, & Jiménez, 2009; Verwey & Wright, 2004) and coarticulation (i.e., the effect in a familiar motor sequence of the next on the current movement, Gentner, Grudin, & Conway, 1980; Jordan, 1995; Rumelhart & Norman, 1982; Sosnik, Hauptmann, Karni, & Flash, 2004). Indeed, some researchers found indications for sequence learning in M1 after extensive practice in a task especially reliant on hand postures, which is the finger opposition task (Karni et al., 1998). This M1-S1 activity may be specific for sequences that rely much on hand postures, are practiced extensively, and are eventually carried out at very high rates. However, we cannot exclude the possibility that this S1-M1 activity was caused by the unusual supine position during scanning with the hand on the keyboard on the participant’s lap, which may have required learning new hand postures too.

The activity in the right superior temporal cortex during execution of familiar sequences was not expected either. The right temporal cortex is usually involved in semantic knowledge (Rice, Lambon Ralph, & Hoffman, 2015; Visser, Jefferies, Embleton, & Ralph, 2012), and is usually not active with sequence learning. It was reported to be active though when participants were recognizing unnatural actions (Binkofski & Buccino, 2006), and expert dancers were viewing familiar movement patterns (Calvo-Merino, Glaser, Grèzes, Passingham, & Haggard, 2005). In the DSP task, participants have been found to select motor chunks as a whole (Verwey, 1999), and perhaps this temporal activity reflects the selection of motor chunks via an abstract representation of the movement pattern as a whole (e.g., an event file; Hommel, 2009; Hommel, Müsseler, Aschersleben, & Prinz, 2001).

## Conclusions

The present study demonstrates how a cognitive model can help understand the broad and variable patterns of neural activity that are typically reported with motor sequencing tasks. The observed neural activities were largely consistent with the hypothesis that motor sequence learning involves three sequence execution systems that develop at different rates, and that race to trigger individual elements in the sequence. We consider this study especially important because these results now allow further tests of the interpretations we gave to the obtained neural activities.

## Footnotes

1 We here focus on representations in discrete familiar motor sequences that have a clear start and end. These representations are believed to play a limited role in cycling tasks like the finger opposition and the serial reaction time tasks (Diedrichsen & Kornysheva, 2015; Jiménez, Méndez, Pasquali, Abrahamse, & Verwey, 2011; Verwey & Abrahamse, 2012; Verwey & Wright, 2014). The reason is that in cycling tasks participants continue to rely on selecting individual responses and that the beneficial effect of practice is due to the formation of associations between successively required representations at all processing levels (Abrahamse, Jiménez, Verwey, & Clegg, 2010). While C-SMB acknowledges this type of sequence learning, we here do not pay further attention to associative sequence learning in cycling tasks.

2 SMAproper would be responsible for element order (Cona & Semenza, 2017). As SMA activity is always observed in sequencing studies, we attribute the absence of SMAproper activity here to this area being involved in the control sequences too.
